# Er:YAG laser removal of zirconia crowns on titanium abutment of dental implants: an in vitro study

**DOI:** 10.1186/s12903-022-02427-4

**Published:** 2022-09-12

**Authors:** Pingping Cai, Yingying Zhuo, Jie Lin, Zhiqiang Zheng

**Affiliations:** 1 Fujian Key Laboratory of Oral Diseases, School and Hospital of Stomatology, Fujian Medical University, 246 Yangqiao Zhong Road, Fuzhou, 350002 Fujian People’s Republic of China; 2 Department of Crown and Bridge, School of Life Dentistry at Tokyo, The Nippon Dental University, 1-9-20 Fujimi, Chiyoda-ku, Tokyo, 102-8159 Japan

**Keywords:** Er:YAG laser, Resin cement, Resin modified glass ionomer cement, Zirconia, Titanium abutment

## Abstract

**Background:**

This research aimed to explore feasibility and the time required when erbium-doped yttrium aluminum garnet (Er:YAG) laser as a non-invasive treatment modality to retrieve different thicknesses of zirconia material bonded by two dental cements from titanium implant abutments.

**Methods:**

Prepared 80 titanium blocks (length: 20 mm, width: 10 mm, height: 10 mm) and square zirconia sheets (length: 10 mm) with different thicknesses (1 mm, 2 mm, 3 mm, and 4 mm) were 20 pieces each. Resin modified glass ionomer cement (RelyX Luting 2; RXL) and resin cement (Clearfil SA luting; CSL) were used to bond zirconia sheet and titanium block. Specimens were kept in 100% humidity for 48 h. Er:YAG laser was used to retrieve the zirconia sheet and recorded the time. Universal testing machine was used to measure the residual adhesion of the samples that did not retrieve after 5 min of laser irradiation. Shear bond strength (MPa) and the time data (s) were analyzed using Kruskal–Wallis Test. The bonding surface and the irradiation surface of the zirconia sheet was examined with the scanning electron microscopy (SEM).

**Results:**

Within 5 min of laser irradiation, RXL group: 1 mm group all fell off, 2 mm group had 3 specimens did not fall off, there was no statistical difference in the average time between the two groups; CSL group: half of the 1 mm group fell off. Shear bond strength test results: there was no statistical difference between 1 and 2 mm in RXL group and 1 mm in CSL group, there was no statistical difference between 3 mm in RXL group and 2 mm in CSL group, and there were significant differences statistically in comparison between any two groups in the rest. SEM inspection showed that the bonding surface and the irradiation surface of the zirconia sheet had changes.

**Conclusion:**

In this vitro study, the following could be concluded: it is faster to remove zirconia crowns with thickness less than 2 mm from titanium abutment when luted with RelyX Luting 2 compared to Clearfil SA luting.

## Background

Increasing patient’s demands and expectations in esthetics have driven the modern dental practice into all-ceramic restorations, zirconia restorative materials due to its excellent mechanical property and biocompatibility have become one of the most used materials in restorative dentistry [1, 2]. Zirconia crowns are usually bonded with resin modified glass ionomer cement or composite resin adhesive on titanium abutments [3, 4]. Dental implants require long-term maintenance, due to restoration damage, food impaction, occlusal adjustment, loosening of abutment screws, treatment of peri-implantitis etc., implant crowns and abutments may need to be removed for replacement or maintenance [5]. Currently, cutting the crown off with using the rotary instrument is perhaps the most used method in clinical, however, this method is difficult and time consuming, and leaves the zirconia crown un-reusable.

Erbium-doped yttrium aluminum garnet (Er:YAG) laser with a wavelength of 2940 nm, theoretically, the wavelengths of these lasers operate with the mid-infrared spectrum, which coincides with the range for water absorption spectrum, the transferred energy can efficiently act on the molecules of water or related groups and is rarely absorbed by other molecules, so that it can precisely act on the water-containing substances to avoid damage to other substances [6–9]. It mainly activates water and monomer molecules in cement between the abutment and crown, these molecules absorb the wavelength and release energy to destructive the polymerized structure of the cement. Based on existing research, it is known that Er:YAG laser can be used to debond orthodontic brackets [10], veneer restorations [11], crowns from natural teeth [12], lithium disilicate crowns from titanium and zirconia abutments [13, 14], and do not cause damage to the dental pulp, abutments or crowns. However, the use of Er:YAG laser to remove cemented zirconia crowns off titanium abutments is yet to be explored.

The aims of this study were to examine the feasibility of a non-invasive retrieval of zirconia crowns from titanium implant abutments by Er:YAG laser in vitro experiment, comparison of the removal effect of two cements and different thicknesses of zirconia materials. The hypothesis was that it was no difference of removal time between composite resin adhesive and resin modified glass ionomer cement. It was also hypothesized that there was no difference between required time from 1 to 4 mm of zirconia material, to provide reference for restoration removal from implant abutment clinically.

## Methods

### Specimen preparation

**Fig. 1:**
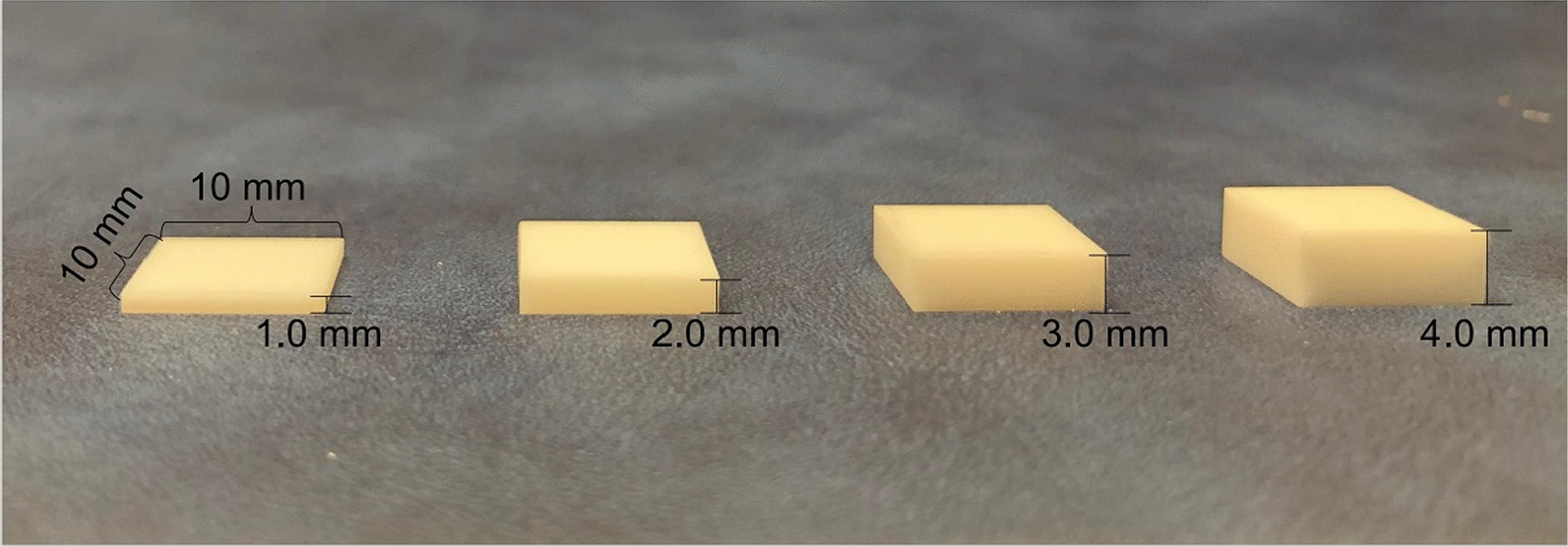
Zirconia sheets

The specimen preparation for the experiment was adapted from methodology by Barbara et al. [15]. In this study, a commercially available yttria-stabilized, tetragonal zirconia polycrystal (Y-TZP) brands-Superfectzir (Aidite Qinhuangdao Technology Co, China) was used. It was a monolithic, translucent and high strength dental zirconia. It was suitable for monolithic crowns. Prepared 80 identical titanium blocks (length 20 mm, width 10 mm, height 10 mm, Preface abutment, Medentika, Germany) and square zirconia sheets (length 10 mm, Superfectzir) with different thicknesses (1 mm, 2 mm, 3 mm, and 4 mm) were 20 pieces each (Fig. 1), which were manufactured from the same materials that are used in implant dentistry. Sand each titanium block sequentially with 320-grit and 600-grit metallographic sandpaper on the automatic polish-grinding machine. The zirconia sheets were airborne particle abraded with aluminum oxide particles with a diameter of 110 μm at 2.8 bar pressure for 20 s from the distance of 10 mm, then all the specimens were ultrasonically cleaned with distilled water for 15 min, and dried with compressed air for 1 min. Application mode and chemical composition of the cements are reported in Table 1. The specimens were divided into RelyX Luting 2 (RXL) group and Clearfil SA luting (CSL) group, and in each group were divided into 4 groups according to thickness, each group had 10 zirconia sheets and 10 titanium blocks (n = 10). According to the manufacturer’s protocol to use the two cements. To standardize the applied pressure, two cements group were bonded under the load of 19.8 N (2 kg) for 5 min. RXL group: then remove excess adhesive; CSL group: the light curing lamp (Elipar S10, 3 M ESPE, USA) irradiated the four sides for 5 s each, removed the excess adhesive, then irradiated the four sides and the top with the light curing lamp for 10 s each. The specimens were then kept in 100% humidity at 37 °C for 48 h.

**Table 1:** List of cements used in this study

Product/code/lot no./manufacturer/cement type	Main composition	Application
RelyX Luting 2/RXL/N748797/3M ESPE (Minnesota, USA) /Resin-modified glass ionomer cement	Paste A: fluoroaluminosilicate glass, proprietary reducing agent, HEMA, water, opacifying agent Paste B: methacrylated polycarboxylic acid, Bis-GMA, HEMA, water, potassium persulfate, zirconia silica filler	Dispense cement onto mixing pad and mix for 20 s, waiting approximately five minutes for the the full self-cure phase
Clearfil SA luting/CSL/0005AA/Kuraray medical (Tokyo, Japan)/Composite resin cement	Bis-GMA, TEGDMA, MDP, barium glass, silica, sodium fluoride	Mix cement through a dual-barrel syringe. Apply, light-cure for 10 s from each side

*HEMA* 2-hydroxyethyl methacrylate; *Bis-GMA* Bisphenol-A-diglycidyl methacrylate; *TEGDMA* Triethyleneglycol dimethacrylate; *MDP* 10-methacryloyloxy-decyl dihydrogenphosphate

## Laser irradiation

The specimens were irradiated by laser Er:YAG (LightWalker; Fotona, Ljubljana, Slovenia) using the R14 handpiece at the following parameters: 300 mJ, 15 Hz, 4.5 W, operation mode: MSP1, spot 0.9 mm, air/water spray at 4/4 with noncontact mode, 1 mm from the surface of the zirconia specimen. Irradiation with the Er:YAG was directed perpendicular to the surface, irradiate on the surface according to the "Z" shape, continuous motion of the laser handpiece on the surface was done to ensure even distribution of laser beam without stagnation, the air/water spray was used throughout the irradiation process. The irrigating solution was distilled water, 20 ml/min. During the irradiation process, if the zirconia specimen was debonded, the total irradiation time was recorded. If the zirconia did not come off after 5 min of irradiation, the specimen was subjected to a shear bond strength test. All experiments were done by the same operator.

## Shear bond strength test

**Fig. 2:**
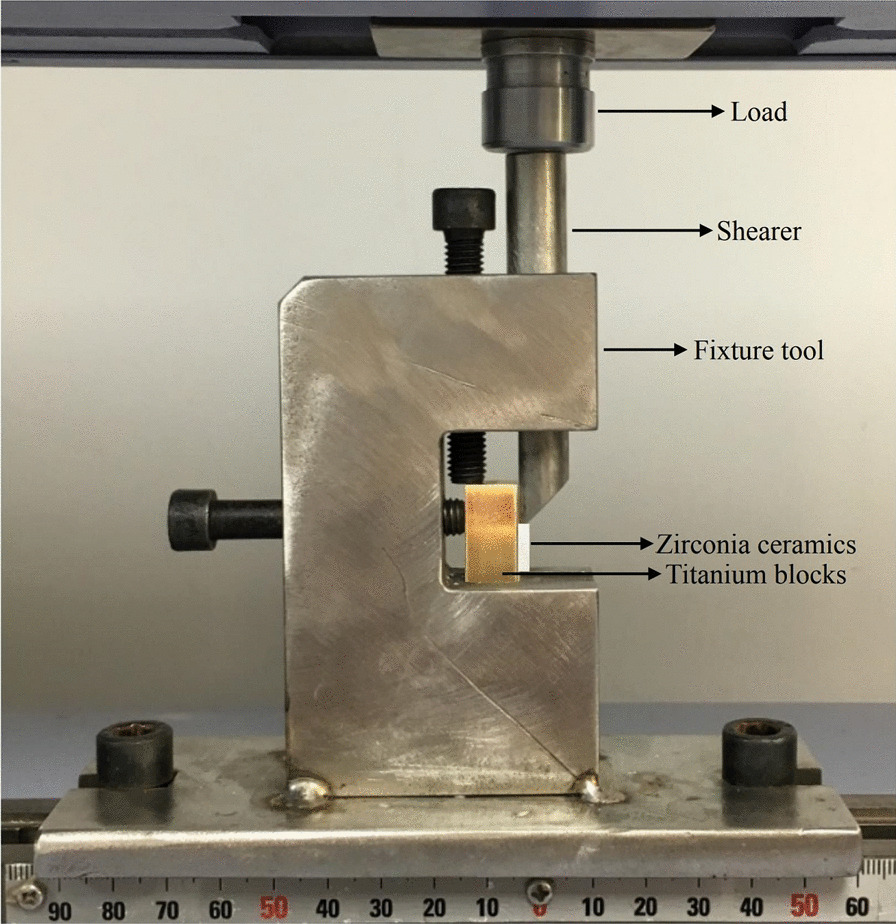
Test jig schematic illustration for determination of shear bond fracture load

The test specimen was placed on the universal testing machine, and the loading head is evenly contacted with the upper edge of the zirconia sheet through the micro-adjustment of the fixture. Shear bond strength was using the Universal Testing Machine (Auto Graph AGS-X500, Shimadzu, Japan) at a crosshead speed of 0.5 mm/min (Fig. 2). The force at separation (N) was divided by the cross-section area (100 mm^2^) to provide results in units of stress (MPa), record the maximum load for debonding. Statistical analysis was performed using the Kruskal–Wallis Test for dismantling time and shear bond strength.

## Scanning electron microscopy (SEM) observation

**Fig. 3:**
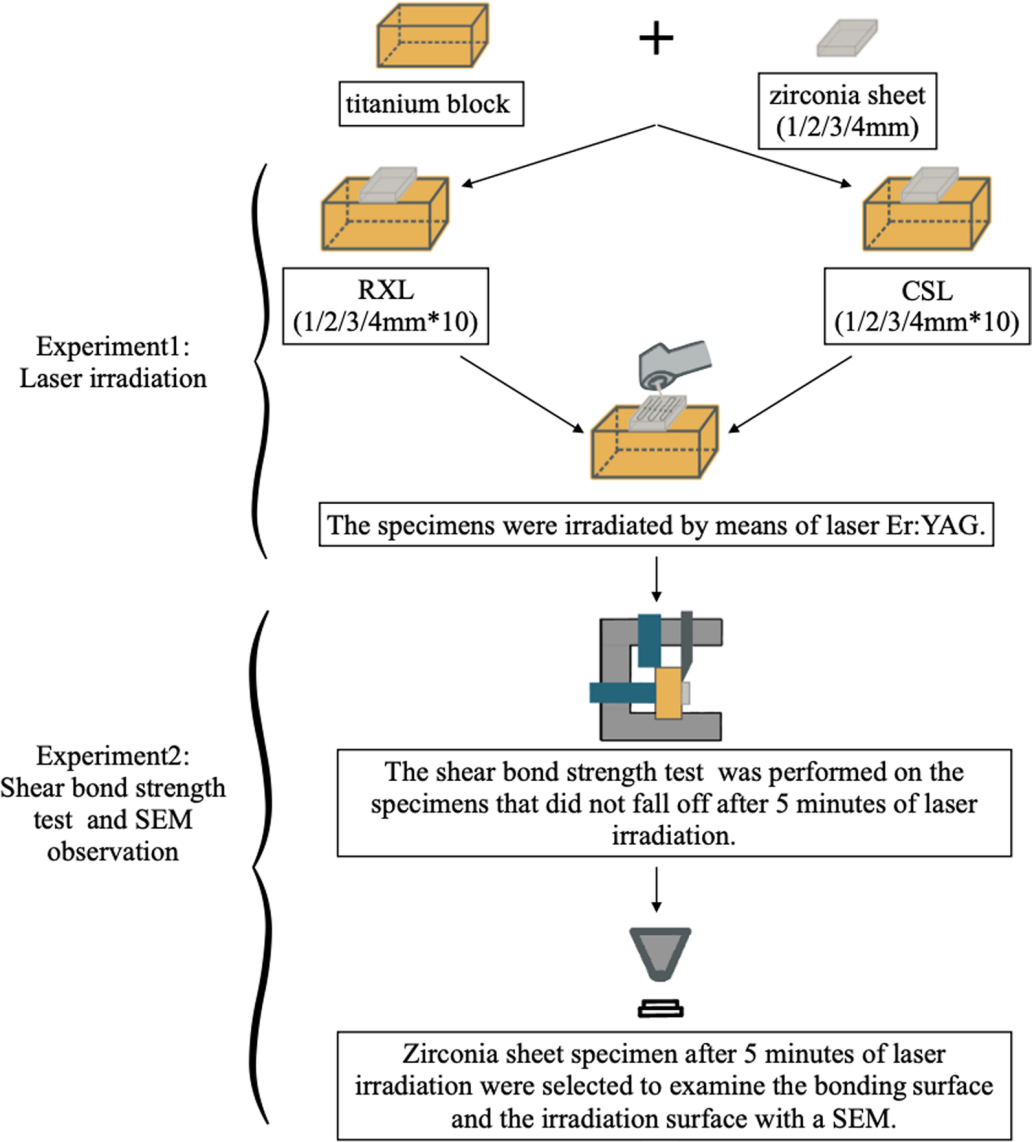
The flow chart of the experimental process

Zirconia sheet specimen after laser irradiation were selected to examine the bonding surface and the irradiation surface with a SEM (NOVA NanoSEM 230, FEI, USA). Representative morphology of the bonding surface of the zirconia sheet were examined in SEM with an acceleration voltage of 5 kV after sputtering using a gold–palladium alloy conductive layer. The flow chart of the experimental process is shown in Fig. 3.

## Results

The Kruskal–Wallis test showed that the comparison results of the removal time and shear bond strength values of the two adhesives and four thicknesses of zirconia sheets are summarized in Table 2 (*P* < 0.05). Within 5 min of laser irradiation, RXL group: 1 mm group all fell off, 2 mm group had 3 specimens did not fall off, the average time for the two groups were 3.3 min and 3.5 min respectively, not statistically different, 3 mm and 4 mm groups did not fall off; the CSL group, only half of the 1 mm specimens fell off, and the average removal time was 4.9 min. For shear bond strength test results, there was no statistical difference between the 1 mm and 2 mm of the RXL group and the 1 mm of the CSL group, and there was no statistical difference between the 3 mm of the RXL group and the 2 mm of the CSL group, there are significant differences statistically in comparison between any two groups.

**Table 2:** Removal time and shear bond strength

		Time (s)		Shear bond strength (MPa)		Removed ratio (%)
Group		Mean ± SD	Median	Mean ± SD	Median	(Proportion of demolished samples)
RXL	1 mm	201.60 ± 47.77	198.00^a^	0 ± 0	0^A^	100
	2 mm	226.50 ± 67.98	213.50^a^	0.71 ± 1.32	0^A^	70
	3 mm	300.00 ± 0	300.00^b^	9.06 ± 3.85	7.66^B^	0
	4 mm	300.00 ± 0	300.00^b^	11.40 ± 4.10	11.22^BC^	0
CSL	1 mm	274.80 ± 35.54	296.50^b^	0.79 ± 1.25	0.26^A^	50
	2 mm	300.00 ± 0	300.00^b^	9.62 ± 4.36	8.88^B^	0
	3 mm	300.00 ± 0	300.00^b^	15.88 ± 4.74	15.07^C^	0
	4 mm	300.00 ± 0	300.00^b^	23.63 ± 8.73	23.33^D^	0

Medians, means and standard deviations (SD) in s and MPa (n = 10)

Medians with the same superscript letter are not statistically different (*P* > 0.05). Kruskal Wallis test followed by pairwise comparison using the Wilcoxon test modified by Bonferroni

**Fig. 4:**
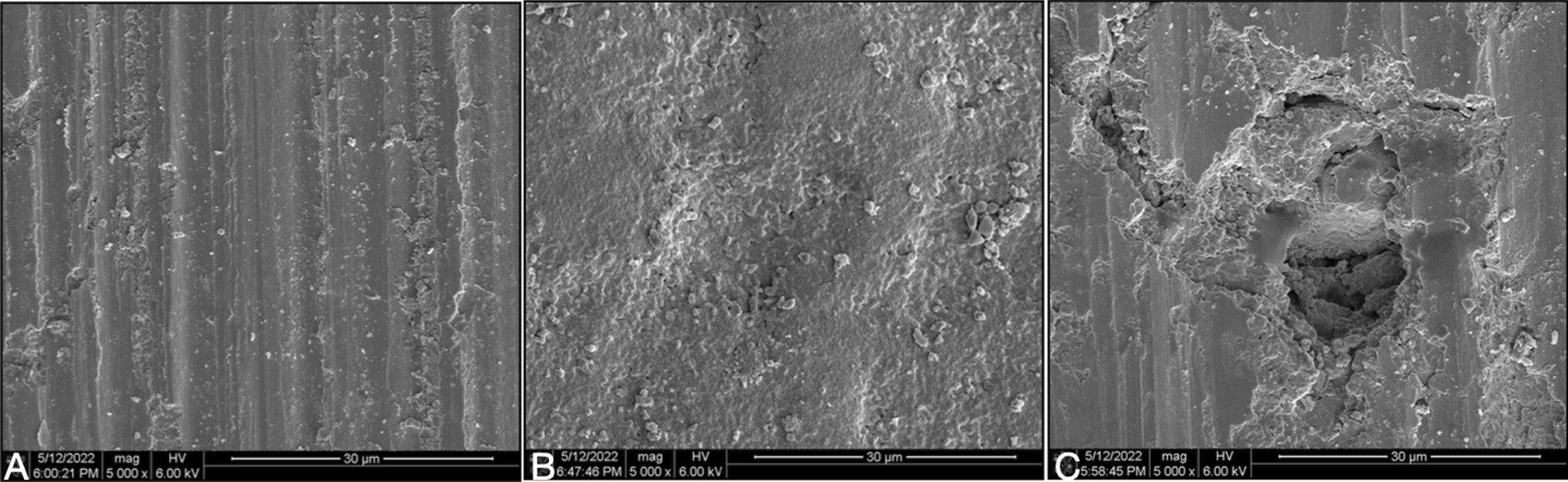
SEM microphotographs (× 5000) of zirconia sheet: **A** Zirconia sheet without laser irradiation; **B** 1 mm zirconia sheet after 120 s of laser irradiation; **C** 3 mm zirconia sheet after 5 min of laser irradiation, burning spots were observed on the surface

**Fig. 5:**
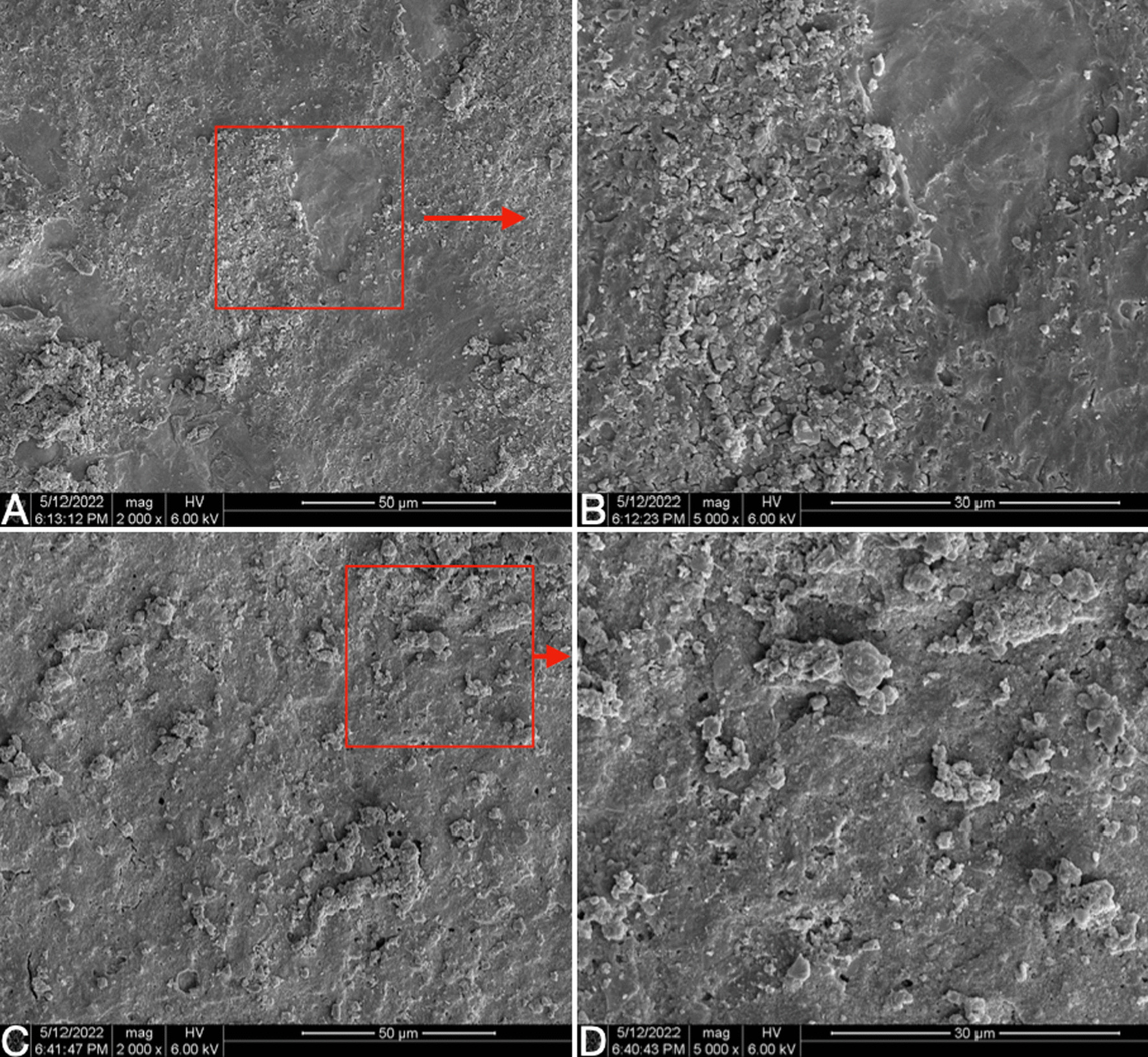
SEM microphotographs of zirconia sheet specimen after 5 min of laser irradiation **A**, **B**: the bonding surface of 2 mm zirconia sheet for RXL group; **C**, **D**: the bonding surface of 2 mm zirconia sheet for CSL group. The burning marks on the surface of the two kinds of cement have different morphologies, and some circular holes can be observed on the surface of RXL

**Fig. 6:**
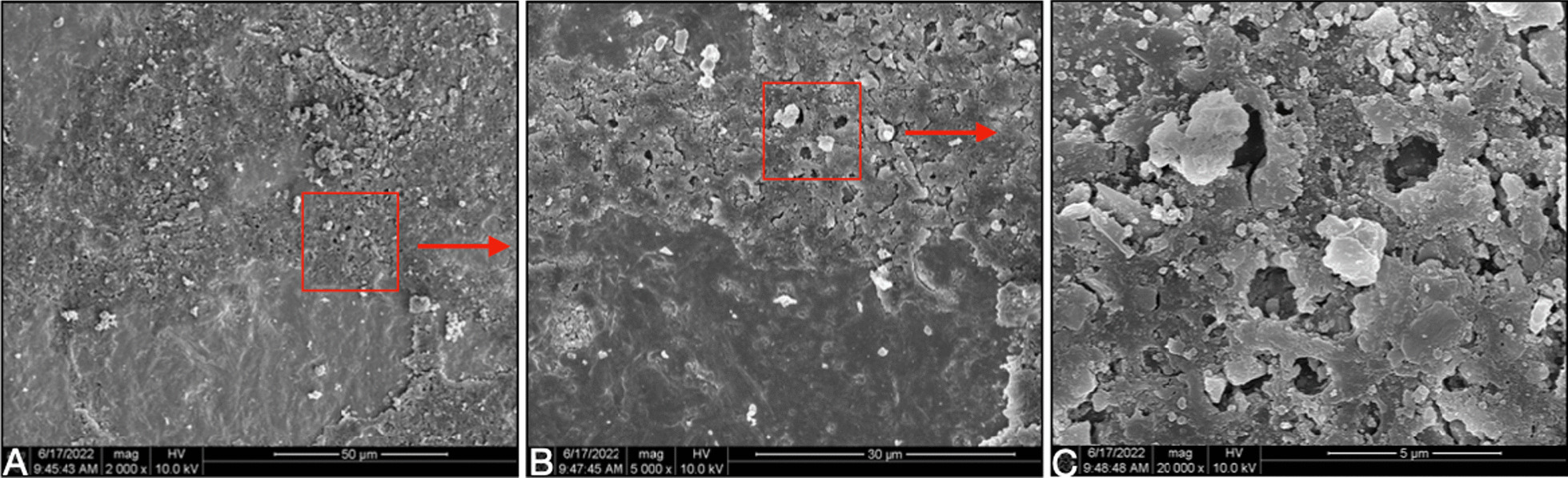
SEM microphotographs of zirconia sheet specimen after 5 min of laser irradiation: the bonding surface of 2 mm zirconia sheet for RXL group. From **A**–**C**, it can be seen that the cement irradiated by the laser has the circular hole like burning marks and rough surface

Visual inspection showed that the zirconia sheets and titanium blocks did not have obvious fractures, and only black burning spots were visible on the surface of some zirconia specimens. SEM observation was performed to test the bonding surface of zirconia sheets. Visual inspection showed that the zirconia sheets and titanium blocks did not have obvious fractures, and black burning spots were visible on the surface of some zirconia specimens. Scanning electron microscope (SEM) observation was performed to test the surface damage of zirconia sheets, compare to the zirconia sheet without any treatment, the inspection showed that the surface of 1 mm or 2 mm with shorter laser irradiation time had minor changes, but the surface of 3 mm or 4 mm with longer laser irradiation time (5 min) had burning pits appeared (Fig. 4). The inspection found that there were obvious fracture points caused by laser on the bonding surface of two groups of laser removes specimen, however, the RXL group had more cracks and more adhesive broken particles than the CSL group (Figs. 5, 6).

## Discussion

In this study, to obtain simpler and standardized data in the experiment, pay attention to the influence of thickness on the laser removal results, and avoid the interference of variables such as abutment size, height, taper, therefore choosing the sheet shape zirconia to instead of the crown shape restoration as the clinical. The follow-up study will focus on the clinical experiment of laser removal of dental crowns. Shear bond strength test results for samples that did not come off after 5 min of laser irradiation. For example, the residual bond strength of 2 mm samples in RXL group and 1 mm samples in CSL group are less than 1 MPa, which can be removed with the aid of clinical tools such as crown remover. However, the residual bond strength of 4 mm samples in RXL group and 3 and 4 mm samples in CSL group are more than 10 MPa, which cannot be removed smoothly. It can be seen that the thickness has a certain impact on the residual bond strength of the samples. Studies have shown [4] among the permanent cements for restorations above implants, the retention force of composite resin cement is greater than that of resin reinforced glass ionomer cement and zinc phosphate cement, this conclusion is consistent with the experimental results.

In addition, the main working principle of the laser to retrieve the restoration is to activate the water and monomer molecules in the adhesive between the crown and the abutment [6–9], this is consistent with the results observed under the electron microscope in this experiment: the bonding surface of 1 mm and 2 mm had numerous adhesive fracture points, the fracture point was circular and was connected to the crack. The ratio of water and monomer components in different adhesives may also affect the absorption of laser energy by the adhesive, thereby affecting the overall dismantling results. Compared with composite resin adhesives, resin-reinforced glass ionomer adhesives contain higher concentrations of water and monomer molecules [14], which can better absorb the energy of laser to destroy the polymeric structure of the adhesive and accelerate the debonding of the restoration.

Previously reported removal of zirconia crowns from natural teeth or implant abutment using an Er:YAG laser was typically 1–2 min (resin-reinforced glass ionomer cement) or about 4–5 min (composite resin cement) [7, 12, 16]. Therefore, in this experiment, 5 min was used as the upper limit time of laser irradiation, and then the shear bond strength test of the adhesive force of the samples that did not fall off was tested to determine whether the laser had an effect. The parameters of the laser in this experiment were set to 300 mJ, 15 Hz, 4.5 W, which is consistent with the previous parameter settings for removing lithium disilicate ceramics from titanium/zirconia abutments and natural teeth [13, 14, 16], and zirconia crowns on the natural teeth were also used this parameter [17]. It can be seen from the existing research reports that this parameter will not cause obvious damage to the tooth and the titanium/zirconia abutment, only the temperature rises, but the temperature change does not damage the surrounding tissue [12]. In this experiment, the scanning electron microscopy observed that minor changes of morphological changes could be seen on the surface of zirconia samples with shorter laser irradiation time, but the appearance of burning pits could be seen on the surface of zirconia samples with longer laser irradiation time. The morphological change may has some impact on the compressive strength of zirconia, but it needs to be further studied in combination with clinical. This experiment is the first time to test whether the Er:YAG laser has a penetrating effect on the 3 mm and 4 mm zirconia sheets, combined with the electron microscope results, it can be seen that after the Er:YAG laser is used to irradiate the 3 mm and 4 mm specimens for 5 min, it seems to have no effect on the adhesive under the specimens.

This in vitro study demonstrated the feasibility of Er:YAG laser to retrieve zirconia crowns from the titanium abutments, however, it still has some limitations. First of all, the zirconia sheets and titanium blocks which were used in this experiment are different from the crowns and abutments used in actual clinical work, and more time may be required to retrieve a crown in the clinical application; secondly, in this vitro study, the laser can better contact the surface of the restoration, avoiding the obstruction of soft and hard tissues (such as adjacent teeth and gingiva), so that the laser can better act on the surface and edge of the restoration; third, in this study, only resin-reinforced glass ionomer adhesives (RXL) and composite resin-based adhesives (CSL) were used, and the proportions of water and monomer molecules in different adhesives are different, the resulting demolition results will also be different; fourth, with the rapid development of zirconia materials, many zirconia materials have appeared clinically, different zirconia materials may have different reactions to laser retrieval due to their different physical properties (such as light transmittance); fifth, the skill of the operator affects the success rate and time required for irradiation. Finally, it is important to note that future clinical in vivo studies with varieties of abutment/crown materials, cements and prosthetic designs, and clinician skills will be needed to further optimize and understand the clinical applications of Er:YAG laser.

## Conclusions

Within the limitations of this in vitro experimental study, it can be concluded that:

Atraumatic decementation of a cement-retained implant prosthesis using Er:YAG laser is a viable method for debonding zirconia crowns from titanium abutments. Er:YAG laser removal is recommended for zirconia crown with thickness less than 2 mm, and resin-reinforced glass ionomer cement is easier to remove than resin cement.

## Data Availability

The complete data and materials described in the research article are freely available from the corresponding author on reasonable request.

## Abbreviations

Er:YAG: Erbium-doped yttrium aluminum garnet
RXL: Resin modified glass ionomer cement
CSL: Composite resin cement
SEM: Scanning electron microscopy
CAD/CAM: Computer-aided design/computer-aided manufacturing
